# Plant–Microbiome Interactions in Medicinal Plants: A Synergistic Partnership for Biomass Production and Secondary Metabolite Accumulation

**DOI:** 10.3390/microorganisms14081650

**Published:** 2026-07-28

**Authors:** Erjun Wang, Yalong Zhang, Ronghua Yue, Yuanyuan Wang, Xiaohui Ma, Gaosen Zhang, Ling Jin

**Affiliations:** 1 College of Pharmacy, Gansu University of Chinese Medicine, Lanzhou 730000, China; wej2024@163.com (E.W.); 15293235407@163.com (Y.Z.); zyxyyrh@163.com (R.Y.); gswyy@gszy.edu.cn (Y.W.); zyxymxh@163.com (X.M.); 2 Northwest Institute of Eco-Environment and Resources, Chinese Academy of Sciences, Lanzhou 730000, China

**Keywords:** medicinal plants, plant microbiome, SynComs, secondary metabolites, co-evolution

## Abstract

Medicinal plants are important sources of secondary metabolites (SMs), but their production is constrained by resource shortages, low cultivation efficiency, and continuous cropping obstacles. As the “second genome” of host plants, the plant microbiome is deeply involved in plant growth and development, stress adaptation, and the accumulation of bioactive compounds, providing new pathways for the sustainable utilization of traditional Chinese medicine resources. This review summarizes the mechanisms by which the plant microbiome regulates biomass formation and SM accumulation in medicinal plants. Microorganisms can promote plant nutrient acquisition, enhance resistance to biotic and abiotic stresses, and regulate root architecture and hormonal signaling. Meanwhile, microorganisms can also participate in the remodeling of secondary metabolic networks in medicinal plants through elicitor- and effector protein-mediated signal transduction, regulation of metabolic gene expression, redistribution of photosynthetic carbon sources and metabolic precursors, and their own biosynthetic capacities. From the perspective of co-evolution, plants and their microbiomes constitute symbiotic systems formed through long-term interactions. Plants can selectively recruit specific microbial taxa through root exudates, SMs, and signaling molecules, whereas microorganisms influence plant adaptability and medicinal material quality through colonization, metabolic feedback, and horizontal gene transfer. This review proposes that a synergistic regulatory pattern of “close phylogenetic relatedness-similar secretions-similar microbial communities” may exist between medicinal plants and microorganisms. This pattern suggests that closely related medicinal plants may share similar core microbial taxa, which may help reveal the intrinsic mechanisms underlying specific microbial recruitment and the quality formation of geo-authentic medicinal materials. Furthermore, the design of synthetic microbial communities (SynComs) can be achieved based on the identification of shared functional genes and the screening of indigenous core functional strains.

## 1. Introduction

Medicinal plants are wild or cultivated plants with recognized medicinal value that can be used for disease prevention and treatment [1]. They also serve as important sources of secondary metabolites (SMs) and precursors for pharmaceutical development, thereby contributing substantially to human health and drug discovery. Traditional medicine is widely used worldwide, and medicinal plants are an important component of many traditional health-care systems [2]. Furthermore, Various bioactive constituents derived from medicinal plants have significant clinical value, such as Ginsenoside Rg3, paclitaxel, artemisinin and breviscapine. These components have been widely used in the intervention of anti-tumor, anti-malaria, anti-fibrosis, and cardiovascular, cerebrovascular and nervous system diseases [3,4]. However, as demand for medicinal plants for the preparation of drugs, phytodrugs, or food supplements continues to grow, issues related to their production efficiency and sustainability have become increasingly prominent [5]. Most wild medicinal plants are endangered due to habitat loss and over-exploitation. Traditional field cultivation faces challenges such as low yield and strong environmental dependence, making it difficult to meet market demands [6].

In recent years, studies on the plant microbiome (including rhizosphere microorganisms, endophytes, and phyllosphere microorganisms) have shown that microbial communities colonizing diverse plant tissues, such as roots, stems, leaves, fruits, and seeds, collectively constitute the plant’s “second genome.” Together with their host, these microorganisms form an integrated holobiont that profoundly influences plant growth and development, stress responses, and the biosynthesis of SMs [7]. In particular, endophytes that colonize the interior of healthy plant tissues without causing obvious diseases can directly or indirectly participate in the biosynthesis of a variety of important medicinal components, such as alkaloids, steroids, terpenoids, flavonoids and glycosides [8]. In addition, endophytic bacteria and fungi can promote the growth of medicinal plants, enhance their tolerance to biotic and abiotic stresses, and may stimulate plants to produce new active compounds [9]. Meanwhile, microorganisms themselves are also a treasure trove of novel natural products. A large amount of evidence shows that natural products derived from microorganisms have immunomodulatory effects [10]. For example, aureusimine and microansamycin isolated from endophytes of medicinal plants have shown potential against the SARS-CoV-2 virus [11]. Alternariol-(9)-methyl ether and alternariol produced by the endophytic fungus (*Pleospora tarda*) of medicinal plants, as well as Cytonic acid A and Cytonic acid B extracted from the fermentation culture of the endophytic fungus *Cytonaema* sp. of European oak, have been confirmed to have the effects of inhibiting viral protease activity and blocking viral replication [12]. Plants and microorganisms have formed specific interaction networks during long-term co-evolution. Among them, a subset of the core microbiota stably colonizes in the rhizosphere or the plant’s interior, which has significant impacts on plant germination, survival, biomass accumulation, flowering time and even overall adaptability [13]. This symbiotic relationship enables plants to effectively adapt to environmental stress, enhance nutrient absorption, resist pathogen invasion, and ultimately regulate the synthesis and accumulation of medicinal components [14].

Based on these insights, this review focuses on the following question: within specific host and environmental contexts, how do plant-associated microbial communities coordinately regulate biomass production, stress adaptation, and the accumulation of SMs in medicinal plants? To address this question, we link microbiome assembly and ecological filtering with microbe-mediated nutrient acquisition, root architecture regulation, phytohormone and immune signaling, metabolic reprogramming, and secondary metabolite biosynthesis. In addition, we further examine the context dependence and limitations of plant–microbe interactions, synthesize contradictory findings, and discuss how a deeper mechanistic understanding may guide the rational design of synthetic microbial communities and the sustainable cultivation of medicinal plants.

## 2. Core Mechanisms by Which Microorganisms Promote the Growth of Medicinal Plants

**Figure 1:**
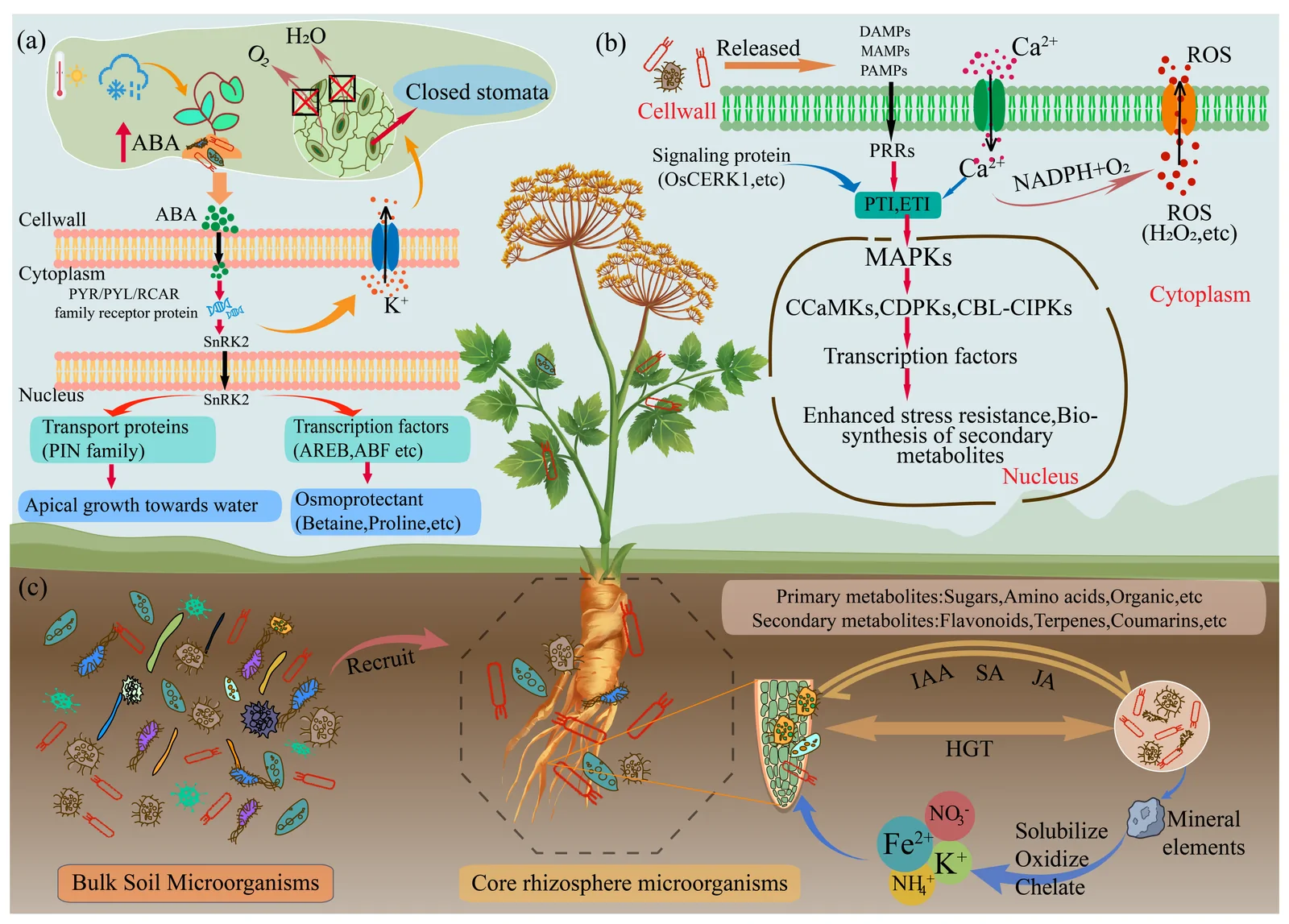
Microbe-mediated shaping of stress adaptation, immunity, and rhizosphere co-evolution in medicinal plants. (**a**) Drought stress-induced ABA biosynthesis in plants and associated microbiomes; (**b**) Pattern-triggered and effector-triggered immune signaling in plant–microbe interactions; (**c**) Rhizosphere microbiome recruitment and horizontal gene transfer for enhanced plant adaptation. Abbreviations: PYR, pyrabactin resistance; PYL, PYR1-like; RCAR, regulatory component of ABA receptor; SnRK2, SNF1-related protein kinase 2; PIN, PIN-FORMED protein; AREB, ABA-responsive element-binding protein; ABF, ABA-responsive element-binding factor; OsCERK1, Oryza sativa chitin elicitor receptor kinase 1; ROS, reactive oxygen species; NADPH, reduced nicotinamide adenine dinucleotide phosphate; MAPKs, mitogen-activated protein kinases; CCaMKs, calcium- and calmodulin-dependent protein kinases; CDPKs, calcium-dependent protein kinases; CBLs, calcineurin B-like proteins; CIPKs, CBL-interacting protein kinases; IAA, indole-3-acetic acid; HGT, horizontal gene transfer.

The interaction between microorganisms and medicinal plants plays a crucial role in their growth and development as well as the formation of medicinal material quality, and this interaction relies on the comprehensive action of multiple synergistic mechanisms (Figure 1). To deeply understand and effectively utilize these mechanisms, we need to establish a systematic interaction framework to clarify how microorganisms support the growth, metabolism, and stress resistance of medicinal plants through specific pathways.

### 2.1. Facilitating Nutrient Acquisition and Cycling

Plants can selectively enrich and assemble a stable core rhizosphere microbiome, thereby promoting the mobilization of soil minerals and the release of nutrients, increasing nutrient availability, and enhancing nutrient uptake and utilization. In medicinal plants, microbe-mediated nutrient acquisition and utilization not only influence plant growth and development but also regulate the biosynthesis and accumulation of SMs (Figure 1c) [15]. Among these nutrients, nitrogen is an essential precursor for the biosynthesis of amino acids, alkaloids, and other nitrogen-containing compounds, and its availability is closely associated with the biosynthesis of bioactive constituents in medicinal plants. However, atmospheric nitrogen exists predominantly as chemically stable N_2_, which cannot be directly utilized by plants [16]. To alleviate nitrogen limitation, plants recruit diverse nitrogen-fixing microorganisms through root-derived signaling compounds and exudates. In legumes, flavonoids mediate specific interactions with rhizobia, promoting root colonization and nodule formation [17]. In addition to symbiotic rhizobia, free-living and associative nitrogen-fixing bacteria, such as *Azotobacter*, *Azospirillum*, and *Herbaspirillum*, inhabit the soil, rhizosphere, or plant tissues and contribute to nitrogen acquisition in both leguminous and non-leguminous plants [18,19,20]. Through biological nitrogen fixation, these microorganisms reduce N_2_ to NH_3_, which is subsequently converted into NH_4_^+^ that can be assimilated by plants; some nitrogenous products may also be transformed into NO_3_^−^ through soil nitrification [21]. In addition, certain rhizobia can invade host roots and induce the formation of root nodules, establishing stable symbiotic associations with plants and thereby enabling more efficient biological nitrogen fixation and nitrogen supply [22]. Co-inoculation of rhizobia and arbuscular mycorrhizal fungi (AMF) in alfalfa increased aboveground and belowground biomass by 88.54% and 236.96%, respectively [23].

In addition to contributing to nitrogen acquisition, soil microorganisms can improve plant phosphorus nutrition through phosphate solubilization, and approximately 20–40% of soil microorganisms possess varying degrees of phosphate-solubilizing capacity [24]. For example, *Pseudomonas fluorescens*, *Bacillus licheniformis*, and *Burkholderia cepacia* can secrete organic acids, such as gluconic and lactic acids, which dissolve insoluble mineral phosphates by lowering the pH and chelating metal ions. Some phosphate-solubilizing microorganisms also secrete phosphatases that promote the mineralization of organic phosphorus into inorganic forms available for plant uptake [25]. Phosphate-solubilizing bacteria likewise show considerable potential in medicinal-plant cultivation. For instance, the combined inoculation of *Pseudomonas putida* and *Pantoea agglomerans* improved vegetative growth, photosynthetic pigment contents, antioxidant enzyme activities, and essential-oil yield in peppermint (*Mentha piperita* L.), while also enhancing phosphorus-fertilizer use efficiency [26]. Most of the iron in the soil exists in the form of Fe^3+^, which cannot be directly absorbed, resulting in extremely low bioavailability [27]. Certain plant growth-promoting bacteria (PGPB) can efficiently chelate Fe^3+^ by secreting iron transporters, converting it into soluble forms and enhancing iron bioavailability [28]. Fungal-derived siderophores also possess a high capacity for efficient iron activation. For example, rhizoferrin synthesized by *Rhizopus arrhizus* can rival synthetic chelators in terms of efficiency in promoting plant iron uptake [29].

Notably, symbiotic microorganisms can supply up to 80% of the nitrogen and 75% of the phosphorus required by plants. Some plant species even rely entirely on microorganisms to complete their life cycles [30]. For example, *Gastrodia elata* seeds lack endosperm, and their entire process from germination to tuber formation depends on the nutrient supply of *Mycena* fungi and the symbiotic relationship with *Armillaria mellea* [31].

### 2.2. Enhancing Resistance to Biotic and Abiotic Stresses

During the long-term coevolution of plants and microorganisms, pattern-recognition receptors (PRRs) located on the plant cell surface recognize damage-associated molecular patterns (DAMPs), microbe-associated molecular patterns (MAMPs), and pathogen-associated molecular patterns (PAMPs), thereby activating pattern-triggered immunity (PTI) [32,33]. Meanwhile, pathogen effectors can be recognized by intracellular nucleotide-binding leucine-rich repeat (NLR) immune receptors, resulting in the activation of effector-triggered immunity (ETI). Extensive signaling crosstalk and mutual potentiation occur between PTI and ETI (Figure 1b) [34,35]. These immune responses induce Ca^2+^ influx, reactive oxygen species (ROS) bursts, and mitogen-activated protein kinase (MAPK) signaling cascades, which subsequently promote the expression of defense-related genes and the biosynthesis of SMs, ultimately enhancing plant resistance to pathogen infection (Figure 1a) [36,37].

In terms of resistance to biotic stress, multiple rhizospheric and endophytic microorganisms can synthesize active substances such as antibiotics, siderophores, cell wall degrading enzymes, or toxins, which act directly on pathogens [38,39]. For example, *B. pumilus* GZDF3 isolated from the rhizosphere of *Pinellia ternata* exerts antifungal effects by secreting siderophores [40], while *Phomopsis cassiae*, a fungus symbiotic with *Senna spectabilis*, can produce cadinane sesquiterpenoids, which significantly enhance the host’s tolerance to a variety of phytopathogenic fungi [41]. Some beneficial microorganisms can also form biofilms on plant root surfaces, effectively crowding out pathogenic bacteria through competition for ecological niches and nutrients [42]. For example, One-year-old *Panax notoginseng* seedlings were inoculated for five months with a mixed AMF inoculum comprising *G. intraradices* and *G. etunicatum* at a 1:1 ratio, resulting in an average root colonization rate of 62.4%. Compared with the non-inoculated control, AMF inoculation increased plant height, root fresh weight, and shoot fresh weight by 31.69%, 55.46%, and 71.53%, respectively. Meanwhile, the contents of notoginsenoside R1, ginsenosides Rg1, Re, Rb1, and Rd, and total *P. notoginseng* saponins increased to 1.85-, 1.56-, 3.47-, 1.64-, 1.94-, and 1.68-fold those of the control, respectively [43].

In addition to direct effects, beneficial microorganisms can also act as elicitors, activating signaling pathways such as jasmonic acid (JA) and salicylic acid (SA) in plants and inducing systemic resistance (ISR), thereby keeping the plants in a “pre–warning” state [44]. For instance, *B. velezensis* enhances plant resistance to gray mold disease by activating the NPR1 and PR1 genes in the SA signaling pathway [45]. Some AMF can transmit signals to uninfected host plants of the same species through the symbiotic hyphal network, thereby alleviating pathogen stress [46]. Moreover, microorganisms can enhance the activities of plant defense enzymes, such as polyphenol oxidase and catalase [47], and promote the synthesis of pest- and disease-resistant compounds (e.g., alkaloids and phenols). This not only alleviates biotic stress but also directly or indirectly facilitates the accumulation of medicinal active components [48].

Microorganisms help plants deal with abiotic stresses like drought, salinity, and heavy metals by enhancing nutrient uptake, regulating physiological metabolism, and aiding in heavy metal detoxification [49,50]. Studies have shown that aquaporins expressed in AMF hyphae can enhance water transport efficiency and contribute to more than 30% of the total water uptake in *Avena barbata*, thereby effectively alleviating drought stress [51]. Moreover, microorganisms can activate antioxidant enzyme systems such as superoxide dismutase, peroxidase, and catalase in plants to promptly remove the excessive reactive oxygen species accumulated under stress conditions, reducing membrane lipid peroxidation and cell damage [52,53]. Meanwhile, microorganisms can also promote the synthesis and accumulation of osmotic adjustment substances such as proline in plants, assist in maintaining the water balance of cells, and thus enhance the adaptability of plants to drought and salt stress [54]. For example, under drought stress, co-inoculation with *G. intraradices* and the plant growth-promoting rhizobacterium *P. putida* increased leaf proline content in fenugreek (*Trigonella foenum-graecum* L.), thereby enhancing osmotic adjustment and alleviating drought-induced damage [55]. Under heavy metal stress, rhizosphere microorganisms function as a “biological barrier,” reducing heavy metal toxicity through mechanisms such as adsorption, precipitation, or transformation [56]. For example, a strain of *Desulfovibrio* sp. SRB1-1 that produces hydrogen sulfide (H_2_S) can immobilize Cd and Pb as metal sulfides with low solubility, reducing the uptake of heavy metals by plants [57].

### 2.3. Regulation of Root Architecture and Phytohormone Signaling

As the main organ for medicinal plants to absorb water and nutrients, the root system’s architectural characteristics (such as root length, root spread, and branching density) directly determine the plants’ efficiency in acquiring resources such as water and mineral elements from the soil [58]. During root development, PGPB mainly promotes root system morphogenesis by synthesizing and regulating plant hormones (Figure 1c). Some PGPB can utilize L-tryptophan in root exudates to synthesize the auxin IAA [22], which in turn induces the expression of genes related to root meristems and promotes the formation of lateral roots and root hairs [59]. Under high-salt stress, *Klebsiella variicola* significantly promoted the adaptability of crop roots by producing IAA [60]. In addition to IAA, certain PGPB strains can also secrete cytokinins, which are involved in regulating apical dominance, cell division, and the senescence process of plants, thereby influencing root development and plant phenotypes [59]. Some PGPB also regulate root growth by producing 1-aminocyclopropane-1-carboxylic acid (ACC) deaminase. This enzyme can break down the ethylene synthesis precursor (ACC) into ammonia and α-ketobutyric acid, thereby reducing the ethylene level in plants under stress and alleviating its inhibition on root elongation [61]. These hormones of microbial origin are directly involved in the regulation of physiological processes such as root development and cell expansion in medicinal plants, ultimately influencing their overall phenotypic establishment.

Unlike the hormonal regulation pathway of PGPB, AMF mainly forms an extensive hyphal network outside the roots, significantly expanding the contact area between the roots and the soil. Meanwhile, it differentiates inside the roots to form arbuscules, which serve as an interface for nutrient exchange with the host. This structure enhances the ability of medicinal plants to absorb elements with poor mobility, such as phosphorus, thereby improving the overall nutrient acquisition efficiency [54]. For example, inoculating AMF can promote the formation of AMF structures in the roots of *Panax notoginseng* seedlings and improve their growth performance [62].

## 3. Microbial Regulation of SMs

Microorganisms are involved in the regulation of plant SMs through various pathways. At the molecular level, elicitors and effector proteins secreted by microorganisms can activate hormone signals such as JA and SA, as well as signaling pathways like Ca^2+^ and MAPK in plants, thereby upregulating the expression of key synthetic genes for SM, such as *HMGR* and *CYP* [63,64]. Meanwhile, Microorganisms can modulate the growth–defense balance of host plants and reprogram primary metabolism, thereby promoting the allocation of photosynthetically fixed carbon and metabolic precursors, such as pyruvate and phenylalanine, toward SMs biosynthesis [65,66]. These multi-level interactions jointly regulate the biosynthesis and accumulation of SMs in plants, thereby contributing to the quality formation of traditional Chinese medicinal materials.

### 3.1. Signal Regulation and Gene Expression

**Figure 2:**
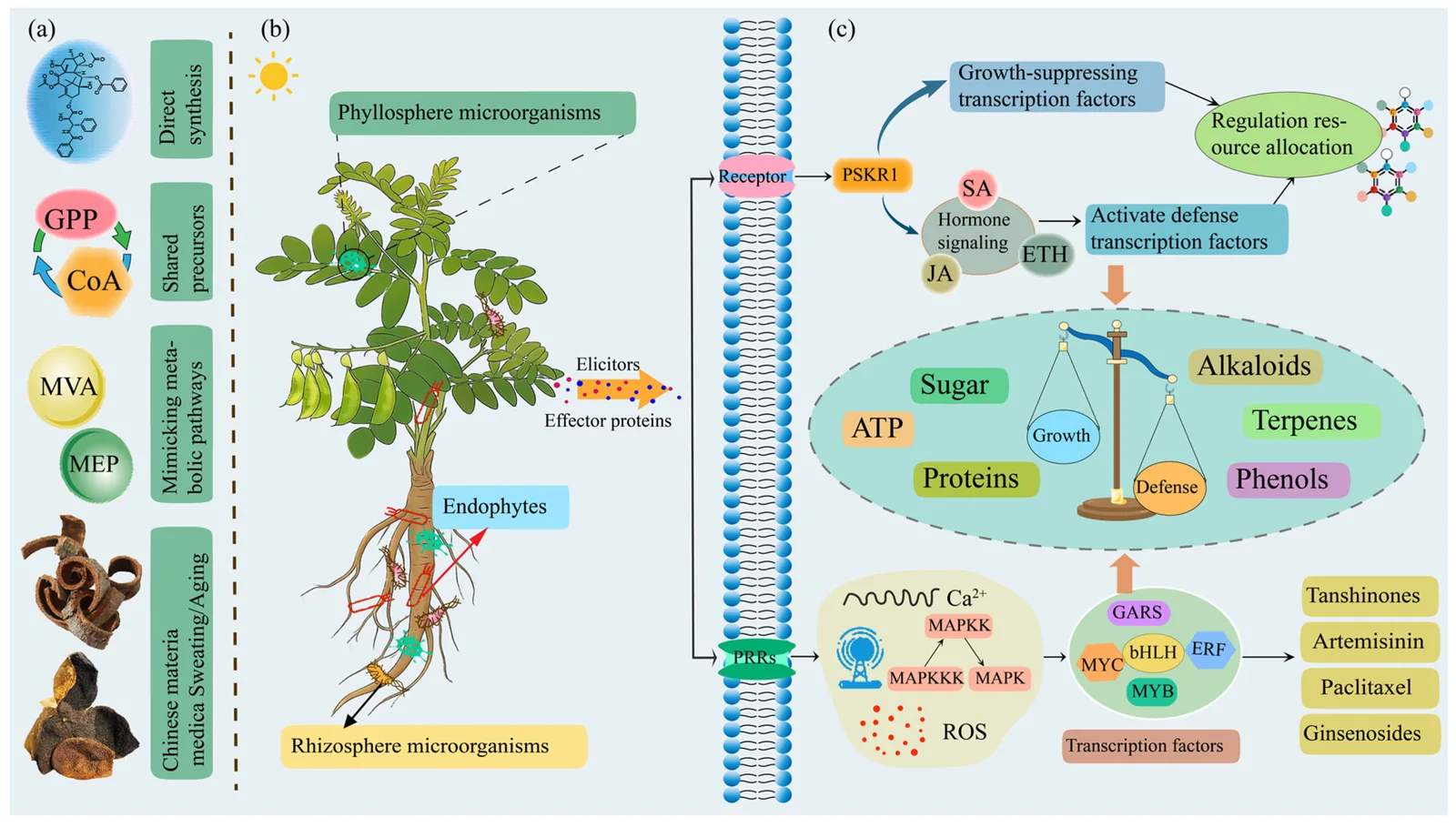
Microbial regulation of SM synthesis and accumulation. (**a**) Synergistic promotion of specialized metabolite accumulation in medicinal plants by associated microorganisms. (**b**) Recognition of microbial elicitors and effectors by plant cell surface receptors in rhizosphere, phyllosphere, and endosphere. (**c**) Signal transduction, transcriptional reprogramming, and growth–defense trade-off triggered by microbial elicitors in plant–microbiome interactions. Abbreviations: GPP, geranyl diphosphate; CoA, coenzyme A; MVA, mevalonate pathway; MEP, 2-C-methyl-D-erythritol 4-phosphate pathway; PSKR1, phytosulfokine receptor 1; ETH, ethylene; ATP, adenosine triphosphate; MAPKKK, mitogen-activated protein kinase kinase kinase; MAPKK, mitogen-activated protein kinase kinase; MAPK, mitogen-activated protein kinase; GRAS, GAI–RGA–and–SCR transcription factor family; MYC, MYC-type transcription factor; bHLH, basic helix–loop–helix; ERF, ethylene-responsive factor.

During the long-term evolutionary process, a complex metabolic interaction network has been formed between plants and microorganisms. Microorganisms can precisely initiate the SM synthesis pathway in plants by secreting elicitors and effector proteins, and profoundly influence the synthesis and accumulation of SMs by regulating relevant signaling pathways and gene expression (Figure 2b) [67]. For example, the extracellular mannan elicitor secreted by *Gilmaniella* sp. AL12 can effectively induce the synthesis of essential oils in *Atractylodes lancea* [68]. In *Atractylodes lancea* plantlets, protein and polysaccharide elicitors isolated from the fermentation broth of the endophytic bacterium *P. fluorescens* ALEB7B stimulated the conversion of terpenoid hydrocarbon scaffolds into oxygenated sesquiterpenoids by triggering an oxidative burst in planta [69]. The action mechanism of these elicitors is closely related to the activation of intracellular signaling pathways in plants. Among them, the calcium ion signaling pathway is considered a key pathway for fungal elicitor-induced synthesis of SMs. Endophytic fungal elicitors were prepared from *Penicillium commune* 2J1 and *Neurospora crassa* 3J1. These elicitors rapidly induced nitric oxide (NO) production in suspension-cultured cells of *Cinnamomum longepaniculatum* and significantly enhanced the accumulation of 1,8-cineole and α-terpineol, with the strongest effects observed at 40 mg L^−1^. An exogenous NO donor further promoted essential-oil accumulation, whereas the NO scavenger cPTIO attenuated the elicitor-induced effects, indicating that NO is an important signaling molecule in endophytic fungal elicitor-mediated essential-oil biosynthesis, although other signaling pathways may also be involved [70].

The regulation of plant SMs by microorganisms is also reflected in the modulation of the host’s endogenous hormone signaling network. Plant hormone signaling pathways, such as those of JA, ethylene (ET), SA, and abscisic acid (ABA), play crucial roles in the synthesis of SMs [71]. Mycorrhizal symbiosis can increase the levels of JA and ABA in plants, induce the up-regulation of the *MYC2* transcription factor, and thereby promote the accumulation of SMs such as anthocyanins and phenolic acids [72]. The fermentation broth of the endophytic fungus *Pseudodidymocyrtis lobariellae* KL27 significantly promoted paclitaxel biosynthesis and accumulation in its host, *Taxus chinensis*, by regulating jasmonic acid signaling and its crosstalk with gibberellin and ethylene signaling pathways [73]. After plants are colonized by endophytes, they often exhibit suppression of the SA signaling pathway, accompanied by an increase in the levels of JA precursors and upregulation of the expression of its responsive genes, creating a signaling environment conducive to the synthesis of specific SMs [74]. In addition, signaling molecules such as hydrogen peroxide (H_2_O_2_) and nitric oxide (NO) can cooperate with various hormones to participate in secondary metabolic regulation [54]. For example, AMF can effectively activate the phenylpropanoid metabolic pathway and promote the synthesis of phenolic compounds by increasing the accumulation of H_2_O_2_, NO, and SA in plants [67,75].

At the gene expression level, the regulation of plant SMs by microorganisms is mainly manifested as the transcriptional regulation of genes encoding key enzymes [54]. Inoculation of *Rhizophagus irregularis* can significantly up-regulate the expression of key genes such as *SQS1*, *β-AS*, *CYP88D6* and *CYP72A154* in the glycyrrhizic acid synthesis pathway, thereby increasing the accumulation of glycyrrhizic acid in *Glycyrrhiza uralensis* [76]. Endophytic fungi can stimulate *HMGR* in the MVA pathway and *DXR* in the MEP pathway. The expression of enzymes such as geranylgeranyl diphosphate synthase (GGPPS), copalyl diphosphate synthase (CPS), and kaurene synthase-like (KSL) in downstream pathways promotes the synthesis of tanshinone [77]. Meanwhile, bacteria also exhibit similar regulatory capabilities. For example, *B. velezensis* NT35 can enhance the expression of genes encoding squalene synthase and dammarenediol synthase in the ginsenoside synthesis pathway [78]. It should be noted that the regulation of plant SMs by microorganisms is not always positively promotive. For example, the mycelial extract of the *Trichoderma atroviride* D16 strain can inhibit the accumulation of phenolic acids in *Salvia miltiorrhiza* [79]. The abundance of *Lysobacter* is significantly negatively correlated with the expression of genes related to the synthesis of liquiritin and glycyrrhizic acid (e.g., *CYP72A154*, *CHI*, *PAL*) [80], reflecting the diversity and complexity of microbial regulatory effects.

### 3.2. Microbially Mediated Resource Reallocation

Plants generally exhibit a “growth–defense” trade-off, in which rapid growth is often prioritized under favorable conditions, whereas defense activation and the biosynthesis of SMs require carbon, energy, and metabolic precursors that might otherwise support biomass production (Figure 2c) [81]. However, this relationship should be regarded as a common but context-dependent tendency rather than a universal or fixed rule, because its direction and magnitude are jointly influenced by host genotype, developmental stage, resource availability, environmental conditions, and crosstalk among metabolic, hormonal, and transcriptional networks [65,81]. Plant-associated microorganisms can modulate this balance by altering nutrient acquisition, phytohormone and immune signaling, photosynthesis, and carbon metabolism [82]. For example, *P. fluorescens* can induce the expression of *PSKR1*, a key regulatory factor in the balance between plant growth and defense, and guide more carbon fixed by photosynthesis to be transported to the roots [83]. *B. amyloliquefaciens* BF1 and *B. subtilis* Y37 can improve the soil microenvironment and nutrient status, and up-regulate the glycolysis/gluconeogenesis pathways. Meanwhile, they significantly promote the bulbing of *Lilium brownii* var. *viridulum* bulbs [84]. Similarly, inoculation with *Glomus mosseae* can enhance the absorption of phosphorus and potassium by the roots of *Glycyrrhiza uralensis*, increasing the root biomass by approximately 17 times and significantly increasing the contents of glycyrrhizic acid, liquiritin, isoliquiritin, and isoliquiritigenin in the taproot [85].

Microorganisms, as natural “metabolic engineers,” can directly or indirectly provide key precursor substances for the synthesis of plant SMs, thereby regulating the synthesis of SMs. The *Streptomyces* Strep-4 can synthesize the terpene precursor GPP, which promotes the accumulation of monoterpenoids in citrus plants [86]. Similarly, the endophytic fungus *Gilmaniella* sp. AL12 can enhance the level of acetyl-CoA (a precursor for sesquiterpene synthesis) in the glycolytic pathway, thereby promoting the accumulation of anaerobic sesquiterpenes in citrus [87]. Microorganisms are also deeply involved in the metabolic regulation of amino acids in plants, providing synthetic precursors for various SMs. For example, *Glomus* can promote the accumulation of tyrosine and phenylalanine in *Ocimum basilicum*, thereby facilitating the synthesis of rosmarinic acid and caffeic acid [88]. The regulation of amino acid metabolism by microorganisms can also be achieved through specific functional genes. Acid phosphatase encoded by the plasmid gene *ppp1* in *P. putida* can promote the absorption of phosphorus and the conversion of phosphoribosyl diphosphate to tryptophan, ultimately promoting the synthesis of pyrroloquinazoline alkaloids in the medicinal plant *Justicia adhatoda* (syn. Adhatoda vasica) [89]. These findings reveal the underlying mechanism of microbial regulation of plant SMs, fully demonstrating the multi-level allocation of host resources by microorganisms as “metabolic engineers”.

### 3.3. Microorganisms Synthesize SMs Endogenously

The plant microbiome colonizes diverse plant tissues and can directly synthesize bioactive compounds or synergistically promote the biosynthesis and accumulation of specialized plant metabolites by supplying shared metabolic precursors, mimicking host metabolic pathways, and employing other mechanisms (Figure 2a) [90]. Studies in the past two decades have gradually revealed that many bioactive components, such as terpenoids, essential oils, phenolic compounds, and alkaloids, are actually derived from plant-associated microorganisms (Table 1). Among plant-associated microorganisms, endophytes have a particularly close association with the SMs of their hosts. They can not only share the precursors for SM synthesis with the hosts but also independently construct their own SM networks by mimicking the metabolic pathways of the hosts [91]. This sharing and mimicking of metabolic capabilities has been verified in various medicinal plants. For instance, the anti-inflammatory activity of *Emilia sonchifolia* can be effectively mimicked by the ethyl acetate extract of its bacterial endophytes [92]. Since the breakthrough report of paclitaxel synthesis by an endophytic fungus from *Taxus brevifolia*, there has been successive evidence indicating that multiple endophytes can synthesize active ingredients identical or highly similar in structure to those of their hosts [93]. For instance, *Fusarium oxysporum* isolated from *Catharanthus roseus* can synthesize vinblastine and vincristine [94]. Among the 132 endophytic fungi isolated from *Nothapodytes nimmoniana*, as many as 94 can produce camptothecin [95].

Microorganisms can profoundly influence the synthesis, transformation, and accumulation of active ingredients through various mechanisms such as metabolic transformation, enzymatic reactions, and community succession, thereby directly regulating the final quality of medicinal materials. During the sweating process of *Magnolia officinalis*, the relative abundances of *Enterobacter*, *Klebsiella*, and *Weissella* are positively correlated with the contents of key pharmacodynamic components such as magnolol and honokiol [109]. It has been suggested that specific microbial groups may contribute to the quality formation of Chinese medicinal materials by promoting the biosynthesis of target compounds. Similarly, during the aging of dried tangerine peel, the microbial community undergoes a distinct successional pattern, with the dominant fungal genus shifting from *Penicillium* to *Aspergillus*. Among them, *Aspergillus niger* shows the ability to efficiently convert precursor substances into polyhydroxy flavones [110].

## 4. From the Perspective of Coevolution and Ecological Adaptability

During the long-term co-evolution of plants and microorganisms, some microorganisms acquire the ability to synthesize plant-specific metabolites through mechanisms such as horizontal gene transfer. Plants can specifically recruit beneficial microorganisms through root exudates, thereby constructing a rhizosphere microbial community with core functional characteristics. The composition and function of this community are not static but show dynamic changes with plant development stages, seasonal alternations, and geographical environments. It directly participates in the formation process of the genuine quality of medicinal plants by regulating the synthesis pathways of SMs.

### 4.1. Horizontal Gene Transfer and Chemical Regulation Mechanisms

Genetic exchange mechanisms, such as horizontal gene transfer (HGT), occur between plants and microorganisms, providing an important genetic basis for their co-adaptation, functional complementarity, and long-term co-evolution. During this process, microorganisms have gradually evolved specific host preferences and colonization patterns, and significantly influenced host metabolism [42,111]. During long-term adaptation to an obligate, host-restricted lifestyle, some plant-associated bacterial endosymbionts undergo substantial genome reduction. For example, the genome of the leaf-nodule endosymbiont “*Candidatus Burkholderia kirkii*” is approximately half the size of those of closely related free-living, plant-associated *Burkholderia* species [66]. This finding suggests that HGT may occur between them during the evolutionary process, and HGT can enable some endophytes to acquire the ability to synthesize plant-specific compounds [112,113]. For example, *F. solani* isolated from *Camptotheca acuminata* can synthesize camptothecin and its derivatives, while strains of the same species from other sources do not have this ability [93]. Similarly, in *Taxomyces andreanae*, the first reported endophytic fungus capable of producing paclitaxel, genes highly homologous to those of the host plant, such as taxadiene synthase and phenylpropanoyl transferase, were also detected [114].

In the context of co-evolution, the composition of root exudates from medicinal plants and the substrate preferences of microorganisms jointly regulate the initial assembly of the rhizosphere microbiome [115]. Root exudates first act as a nutrient source to drive the reorganization of the microbial community. Subsequently, plant genotypes further fine-tune the interaction relationships between plants and microorganisms, resulting in the formation of a highly specific rhizosphere microbial community [116,117]. *Codonopsis pilosula* specifically recruits soil microorganisms by releasing volatile organic compounds (VOCs) such as (2, 3, 5-trimethylpyrazine), and further affects the accumulation of phenylpropanoid metabolites and the expression of related genes [118]. *Artemisia annua* specifically recruits PGPB, such as *Sphingomonas* and *Sphingobium*, by secreting terpenoids like artemisinin [119]. This indicates that, during the long-term coevolution of plants and microorganisms, a group of “core microbiomes” closely associated with plant metabolism has gradually emerged.

Closely related plant species typically secrete similar root exudates, thereby recruiting and maintaining similar microbial community structures. AMF exhibits distinct host-specific colonization patterns at the family taxonomic level [120]. For instance, plants in the Brassicaceae family selectively regulate the colonization of rhizosphere microorganisms by synthesizing SMs such as aliphatic glucosinolates (AGs) and indolic glucosinolates (IGs) [121]. In *Brassica* spp. plants, IGs can be activated by specific β-glucosidases to generate brassinin, thereby enhancing the selection of the rhizosphere microbial community [122]. Similarly, the symbiosis between Fabaceae plants and rhizobia relies on flavonoid signaling molecules secreted by the root system, such as luteolin and 7,4′-dihydroxy flavone. Such molecules can activate rhizobial nod gene expression and promote the synthesis of Nod factors. Subsequently, it is recognized by plant LysM-type receptor kinases (RLKs) and triggers signal cascade conduction, ultimately regulating the formation of root nodulation [123]. Wicaksono et al. compared the bulk-soil, rhizosphere, and root-endosphere microbiomes associated with *Matricaria chamomilla*, *Calendula officinalis*, and *S. distichum* grown in organically managed desert farmland in Egypt. Bacterial richness and diversity progressively declined from bulk soil through the rhizosphere to the root endosphere, indicating that plant roots exert strong ecological filtering on microbial communities. Plant species explained 50.5% and 34.6% of the variation in bacterial community structure in the rhizosphere and root endosphere, respectively, demonstrating that host identity has a particularly strong influence on rhizosphere microbiome assembly. Notably, the two Asteraceae species, *M. chamomilla* and *C. officinalis*, harbored more similar rhizosphere microbial communities, whereas the community associated with the perennial solanaceous plant *S. distichum* was clearly distinct, further suggesting that host phylogeny and life-history traits jointly shape microbiome assembly [13]. In addition, research shows that for *Cynomorium songaricum* growing in two habitats, desert steppe and saline-alkali land, the compositions of their rhizosphere microbial communities are similar, but there are differences in their abundances [124]. Collectively, these research results imply that there might be a coordinated regulatory pattern of “close kinship-similar secretions-similar microbial communities” between plants and microorganisms.

### 4.2. Adaptive Reconfiguration of the Plant Microbiome

Plants can sense various environmental signals such as light, water, touch, herbivores, pathogens, and symbiotic microorganisms, and enhance their ecological adaptability by releasing corresponding signaling molecules [125]. When plants are simultaneously exposed to multiple environmental cues, associated changes in epigenetic regulation, small-RNA-related pathways, and plant metabolism may alter root exudation, immune status, and other host traits, thereby influencing microbiome assembly. These context-dependent changes in microbial community composition may, in turn, contribute to plant stress acclimation and resistance [126]. For example, essential oil plants can form “phytogenic fields” around their growing areas through fallen leaves, effectively limiting microbial diversity and constructing a microecosystem favorable to their own growth [127]. This active reshaping is particularly prominent in the rhizosphere microbiome. Research shows that the rhizosphere region is significantly enriched with functional genes related to nutrient transport, defense responses, and SM synthesis [13]. Microorganisms carrying such specific functional genes further influence plant productivity and ecological adaptability by regulating plant functional traits [128].

The community structure of the plant microbiome is regulated by the plant development cycle and environmental factors, showing significant spatiotemporal heterogeneity [129]. On the temporal scale, the dynamic changes in root exudates drive the regular succession of the structure and function of the rhizosphere microbial community along with different plant growth stages [130,131]. In the early stage of plant growth, nutritional substrates such as organic acids, amino acids, and carbohydrates secreted by roots contribute to the recruitment of beneficial microbial communities such as *Sphingomonas* [132]. These microbial communities can synthesize plant hormones such as IAA, thereby promoting root development. As plants enter the senescence stage, their nutrient absorption capacity weakens, and root exudates gradually shift from primary metabolites to SMs to meet the physiological requirements of plant survival and defense [133]. This shift in metabolites not only enhances plant resistance but also inhibits the growth of beneficial microorganisms and promotes the proliferation of potential pathogens [134]. For example, in the initial stage of root development of *Rehmannia glutinosa*, the nutrient-type secretions facilitate the colonization of *Sphingomonas*, and at this time, the content of the medicinal component forskolin is relatively low. As the roots enter the mature stage and the synthesis of forskolin reaches its peak, *Kaistobacter*, which may be related to forskolin synthesis, becomes the dominant genus. In the late growth stage, as the synthesis of medicinal components weakens, the abundance of *Kaistobacter* decreases, while the microbial communities responsible for organic matter degradation (e.g., *Flavisolibacter*) are relatively enriched [135].

In terms of spatial scale, there are differences in the microbial community structure among different geographical regions, which also affect the accumulation of medicinal components. For example, there are differences in the composition and abundance of endophytic bacteria in the root tissues of *Bistorta vivipara* from different regions, which influence the accumulation of various medicinal components in *Bistorta vivipara* [14]. Furthermore, there are significant climatic zonality differences in the ecological effects of soil microorganisms and their contributions to plant adaptability [136]. Soil microbial communities from dry and warm regions can enhance the drought resistance of plants more effectively than those from cold regions [137].

### 4.3. The Association Between the Microbiome and the Geo-Herbalism of Chinese Medicinal Materials

The geo-herbalism of Chinese medicinal materials may be associated with the ecological adaptability of rhizosphere microorganisms. Several studies have revealed the potential role of microorganisms in SM synthesis and quality formation from different medicinal plants. In *Citri Reticulatae Pericarpium* (dried tangerine peel), *Streptomyces* and *Serratia* have been shown to synergistically promote the accumulation of monoterpenes such as α-pinene, β-pinene, and α-thujene [86]. Similarly, the biosynthesis of sesquiterpenes in *Atractylodes macrocephala* Koidz is closely linked to rhizosphere microbial communities characteristic of geo-authentic product area [138]. Furthermore, the microbial communities of *Dendrobium officinale* differ significantly between the geo-authentic product area and non-geo-authentic areas; Ceratobasidiaceae fungi are notably enriched in geo-authentic product areas and are positively correlated with polysaccharide content [139]. These studies collectively indicate that the unique qualities of geo-authentic herbs not only originate from the plants themselves but are also closely related to the long-term co-evolution of their rhizosphere microbiome.

## 5. Applications and Ecological Principles of SynComs

The microbiome offers new avenues for the production of bioactive compounds in medicinal plants. At the cultivation level, microbial fertilizers and biostimulants can partially replace the use of chemical fertilizers and pesticides, thereby promoting plant growth and alleviating continuous cropping obstacles. Furthermore, the rational design of SynComs based on ecological and omics data, combined with the screening of optimal indigenous functional strains and appropriate inoculation timing, is expected to enable the stable colonization of functional strains and regulate the physiological metabolism of medicinal plants.

### 5.1. Sustainable Cultivation Practices for Medicinal Plants

In recent years, issues such as the excessive use of chemical fertilizers and pesticides and the intensification of continuous cropping obstacles in the cultivation of medicinal plants have led to the imbalance of the rhizosphere microbial community structure and the frequent occurrence of soil-borne diseases [140]. Utilizing Syncom to enhance the adaptability of crops under adverse conditions has emerged as a promising strategy (Table 2). Compared with chemical fertilizers and pesticides, green biological products represented by biofertilizers, biopesticides, and biostimulants have received extensive attention globally in recent years [141].

### 5.2. SynCom Design Based on Ecological Principles

**Figure 3:**
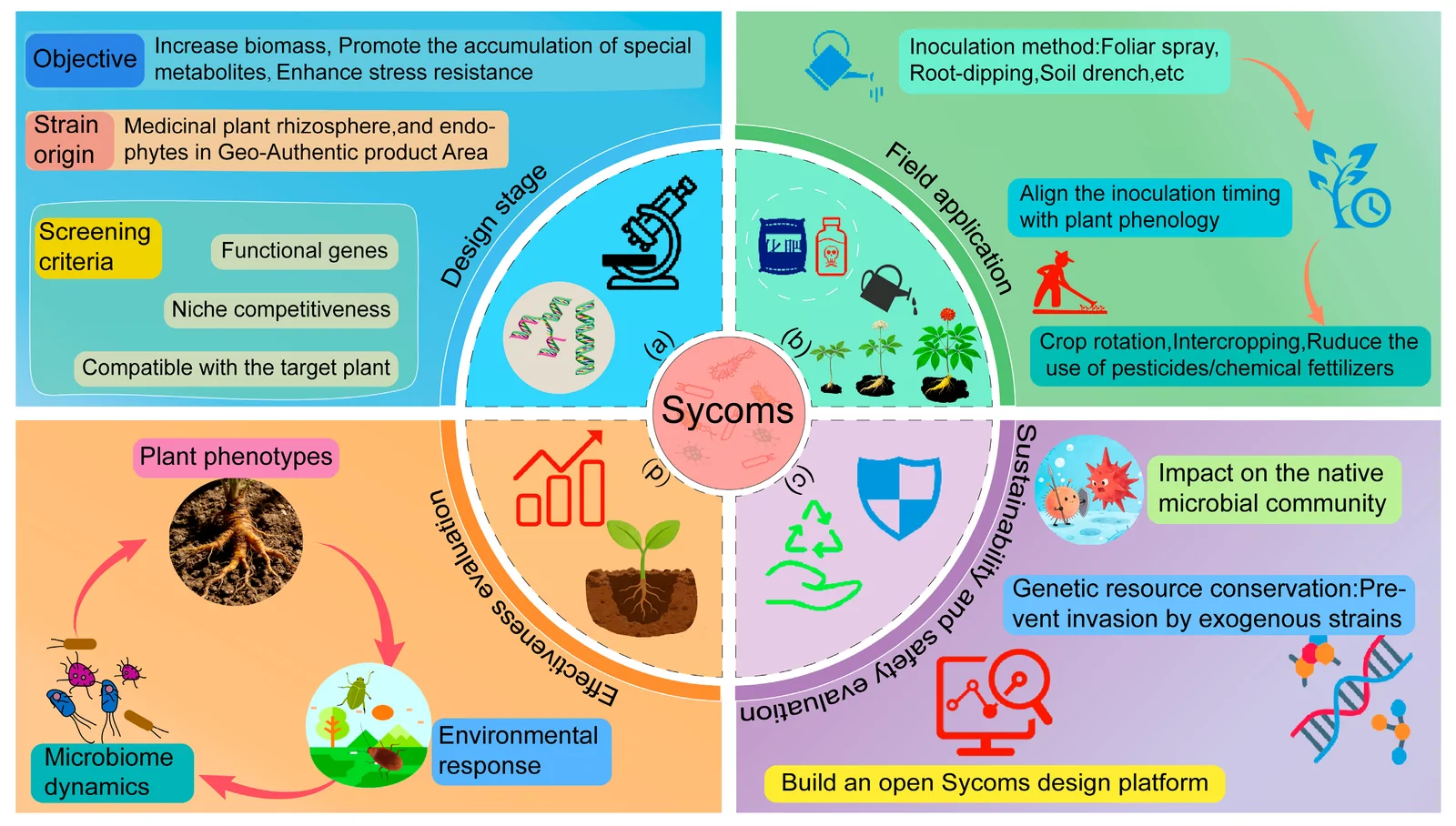
Design principles and applications of SynComs. (**a**) Screening and design of host-compatible Syncoms from medicinal plant microbiomes. (**b**) Flexible inoculation and environmental conditioning for Syncom colonization. (**c**) Multidimensional evaluation of Syncom effects on plants, microbiomes, and environment. (**d**) Ecological safety assessment and data sharing for sustainable Syncom application.

SynComs can be defined as “consortia of microorganisms designed to mimic, at some scale, the observed functions and structure of the microbiome in natural conditions” [155]. It has demonstrated remarkable application potential in aspects such as enhancing soil fertility, ensuring food security, mitigating climate change, and ecological restoration [156]. However, influenced by the complexity of the soil–plant–environment system and the competition of native microbiota, SynComs often encounter problems such as difficult colonization, low survival rate, and unstable functions after being introduced into new habitats [157]. To improve the practical performance of SynComs, their design should integrate multidimensional data from ecology, genomics, and physiology, prioritize indigenous microbial resources, and select microorganisms from the rhizosphere, internal tissues, and other relevant habitats of medicinal plants in geo-authentic production areas based on their phylogenetic compatibility with the host, functional complementarity, and colonization capacity, thereby enabling the construction of stable and efficient SynComs (Figure 3a). Research indicates that indigenous microorganisms not only have a lasting positive effect on soil functions and plant growth (i.e., legacy effect) [158] but also form a “home-court advantage” through their ecological adaptability, colonization ability, and metabolic compatibility, and are generally superior to exogenous strains in practical applications [156].

From the perspective of ecological theory, the biodiversity–function theory provides an important basis for the construction of SynComs. Multi-species combinations can enhance community functions through complementarity and selection effects: the former emphasizes functional complementarity among different species, whereas the latter suggests that higher species richness increases the probability of including key functional taxa within the community [156]. Therefore, SynCom construction should not simply pursue an increase in strain number but should prioritize the selection of dominant strains with specific target functions, strong resource utilization capacity, high niche competitiveness, and good environmental adaptability [159]. When screening core microorganisms, indicators such as phylogenetic diversity, functional gene enrichment, and environmental adaptation phenotypes should be considered to improve the structural rationality and functional stability of SynComs. Meanwhile, the design of SynComs should combine the compositional characteristics of natural microbial communities (e.g., amplicon sequencing data) and integrate multi-omics information, such as metagenomics and metabolomics, to construct synthetic communities with target functional characteristics. In addition, the inoculation timing is crucial for the successful colonization of SynComs and needs to match the plant phenological stages. In particular, precise intervention during the peak nutrient demand period (e.g., the flowering stage) can effectively promote community assembly and functional expression (Figure 3b) [160]. Inoculation at the seedling stage can utilize “priority effects” (the phenomenon in which early-colonizing species influence the colonization and functions of subsequent species through resource competition and niche occupation), enabling SynComs to occupy root surface and rhizosphere niches, avoiding competition from indigenous microorganisms, and laying a foundation for the stable performance of subsequent functions [161,162]. In practical applications, the colonization and functional performance of SynComs should be comprehensively evaluated using multidimensional indicators, alongside ecological safety assessments and the establishment of standardized shared databases for continuous monitoring and risk management, thereby ensuring their safe, stable, and controllable application and promoting the sustainable utilization of medicinal plant–associated microbial resources (Figure 3c,d).

## 6. Current Challenges and Future Perspectives

SynComs have shown considerable potential for application in medicinal plant cultivation; however, their large-scale deployment still faces multiple challenges. More than 99% of environmental microorganisms are difficult to cultivate using conventional methods, severely limiting the discovery and utilization of functional microbial strains [163]. Metagenomics offers a culture-independent solution to this limitation through the reconstruction of metagenome-assembled genomes, enabling researchers to directly characterize the genetic information of uncultured microorganisms and to design targeted cultivation strategies based on their predicted metabolic functions [164]. This approach has been widely applied in studies of gut microbial SynComs and has demonstrated substantial feasibility [165,166], but its application to plant microbiomes, particularly the construction of SynComs associated with medicinal plants, remains relatively limited. Moreover, the field performance of SynComs is often constrained by inconsistent efficacy, strong dependence on plant genotype, and competitive exclusion by indigenous microbial communities, making community behavior difficult to predict [54]. To address these challenges, multidimensional information from genomics, metabolomics, and transcriptomics should be integrated with machine-learning models to elucidate the assembly mechanisms of natural microbial communities, such as environmental filtering and interspecific interactions, as well as adaptive strategies shaped during long-term evolution, including the evolution of stress-resilient phenotypes and the deployment of functional redundancy. Such approaches may enable SynComs to more faithfully recapitulate the key structural and functional characteristics of natural communities, thereby improving their stability and environmental adaptability [10,167]. For example, the “SuperCC” model developed by Ruan et al. successfully constructed an efficient SynCom for the remediation of herbicide-contaminated soil by resolving metabolic interactions within microbial consortia [168]. Of particular interest, the rhizoplane microbiome represents a specialized microbial community that directly contacts the root surface and responds to selection pressures operating across the rhizoplane–rhizosphere interface. It therefore plays a key role in regulating the colonization of root-associated microorganisms [169]. Future research should consequently place greater emphasis on the rhizoplane niche to provide a theoretical basis for functional-strain screening, community-structure optimization, and enhancement of SynCom colonization capacity, thereby advancing the development of next-generation SynComs with greater efficiency, stability, and adaptability.

Although numerous studies have sought to develop SynComs suitable for medicinal plants and have provided preliminary evidence of their potential to promote plant growth, enhance stress tolerance, and regulate the biosynthesis of SMs, research in this field remains at an exploratory stage, and a comprehensive theoretical framework and practical implementation pathway have yet to be established. Based on the current state of research, we propose the following targeted strategies. (1) Multi-omics data should be integrated to elucidate medicinal plant–microbe–environment interactions. Greater emphasis should also be placed on the exploration of indigenous microbial resources and on PGPB shared among phylogenetically related medicinal plant species, with the aim of constructing SynComs that can colonize stably and are compatible with multiple medicinal plant hosts. (2) Microorganisms capable of producing SMs identical or similar to those of their host plants should be screened, and plant-like intracellular microenvironments should be simulated to enable the in vitro microbial production of SMs from medicinal plants. (3) The molecular dialogue between plants and their microbiomes should be investigated in greater depth, and key signaling molecules should be manipulated to enhance the recruitment of beneficial microorganisms by plants. (4) Functional genes shared by microorganisms and their medicinal plant hosts should be identified, and SynComs should be designed according to gene functions and matched to specific stages of the medicinal plant growth cycle. For example, strains carrying functions that synergize with plant nutrient-uptake pathways could be selected for the seedling stage, whereas strains functionally coordinated with secondary-metabolite biosynthetic pathways could be introduced during the root-enlargement stage. This strategy would promote a transition in SynCom construction from the simple combination of microbial strains toward the precise assembly of functionally coordinated consortia based on gene-level complementarity.

## Figures and Tables

**Table 1:** Secondary metabolites from Endophytes and their biological activities.

Medicinal Plants	Endophytes	Secondary Metabolite	Biological Activity	References
*Cyclocarya paliurus* (Batal.) Il jinsk.	*Monascus purpureus*	Exopolysaccharide	antioxidant activity	[96]
*Catharanthus roseus* (L.) G. Don	*Fusarium oxysporum*	Vinblastine, vincristine	Anti-tumor	[94]
*Nothapodytes nimmoniana* (J. Graham) Mabb.	*Alternaria alstroemeriae* NCIM1408, *Alternaria burnsii* NCIM1409	Camptothecin	Anti-tumor	[95]
*Artemisia nilagirica* (C. B. Clarke) Pamp.	*Chromobacterium violaceum* WVAT6, *Burkholderia* sp. WYAT7	Ethyl acetate extract	Antibacterial	[97]
*Artemisia absinthium* L.	*Pseudomonas aeruginosa* SD01	2-aminoacetophenone, phenazine	Antibacterial, Antiviral	[98]
*Aster tataricus* L. f.	*Cyanodermella asteris*	Astin-class	Anti-tumor	[98]
*Taxus cuspidata*	*Penicillium* HDS-Z-1 E	N-acetyl-hydrazinobenzoic acid	Liver protection	[99]
*Dicoma anomala* Sond.	*Pantoea* sp. MHSD15, *Stenotrophomonas* sp. MHSD12	9-Octadecenamide, (Z)	Antioxidative, hypolipidemic	[100]
*Chinese mangrove plant*	*Phomopsis asparagi* DHS-48 and *Phomopsis* sp. DHS-11	3β-hydroxy-5,9-epoxy-(22E,24R)-ergosta-7,22-dien-6-dione	Immunosuppressive	[101]
*Taxus wallichiana var. chinensis* (Pilger) Florin	*Alternaria* sp.	ethyl acetate extracts	Cytotoxic, antimicrobial	[102]
*Solanum nigrum* L.	*Bacillus* sp. MHSD_37	oligopeptides	Anticancer, antimicrobial	[103]
*Cinnamomum cassia* (L.) J.Presl	*Streptomyces cavourensis* YBQ59	bafilomycin D,1-monolinolein	Antibacterial, antitumor antibiotic	[104]
*Mikania micrantha* Kunth in Humb. & al.	*Micrococcus yunnanensis*	microansamycin	Antiviral (SARS-CoV-2)	[105]
*Solanum mauritianum*	*Penicillium chrysogenum*	1,8-Diazacyclotetradecane-2,9-dione A	Antibacterial	[106]
*Dendrobium huoshanense* C. Z. Tang & S. J. Cheng	*Streptomyces* sp. HS-3-L-1	Huoshanmycins	Cytotoxic	[107]
*Rauvolfia verticillata* (*syn. Rauvolfia yunnanensis*)	*Fusarium sporotrichioides*	12a-acetoxy-4,4-dimethyl-24-methylene-5a-cholesta-8,14-diene-2a,3b,11b-triol	Inhibit cancer cells, cytotoxic activity	[108]

**Table 2:** Application examples of PGPB in medicinal plant cultivation.

Medicinal Plants	Syncom	Promoting Mechanism	References
*Adhatoda vasica*	*Azotobacter chroococcum*, *Pseudomonas putida*	Promote the accumulation of pyrroloquinazoline alkaloids	[89]
*Panax ginseng* C. A. Mey.	*Aspergillus niger*	Total ginsenoside content increased by 209.1%	[142]
*Abrus mollis* Hance	*Rhizobium tropici* FM-19	Increased total phenolic and total flavonoid content	[143]
*Dendrobium nobile* Lindl.	Mycorrhizal fungus MF23	Dendrobine increased by 2.88 times	[144]
*Salvia miltiorrhiza* Bunge	*Chaetomium globosum* D38, *Trichoderma atroviride* D16	Promote the accumulation of tanshinone	[145]
*Artemisia annua* L.	*Bacillus subtilis*, *Bacillus licheniformis*, *Burkholderia* sp., *Acinetobacter pittii*	Artemisinin increased by 658%	[146]
*Taxus chinensis* (Pilg.) Rehder	*Micromonospora* sp. TSI4-1	Increase paclitaxel content	[147]
*Papaver somniferum* L.	*Acinetobacter* sp. SM1B, *Marmoricola* sp. SM3B	Increased morphine content	[148]
*Panax ginseng* C. A. Mey.	*Bacillus altitudinis* LB5-3	Saponin (Rg3, Rh1, Rh2) content increased fourfold	[149]
*Platycodon grandiflorus* (Jacq.) A.DC.	*Rhizobium* sp. BF-E16	Increase polyphenol content	[150]
*Rheum palmatum* L.	*Bacillus amyloliquefaciens* EZ99	Increase root fresh weight and active ingredient content.	[151]
*Dioscorea opposita* Thunb. cv. Tiegun	*Bacillus*, *Pseudomonas*, and *Enterobacter*	Solubilize phosphorus and enhance resistance to pathogenic bacteria	[152]
*Andrographis paniculata* (Burm. f.) Wall. ex Nees	*Bacillus gaemokensis* CIMAP-A7	Promote the accumulation of SMs	[153]
*Cnidium officinale*	*Leclercia adecarboxylata*	Suppressing wilt disease	[154]

## Data Availability

No new data were created or analyzed in this study. Data sharing is not applicable to this article.
